# Prescriptions and Dispensing

**Published:** 1908-02-08

**Authors:** 


### Therapeutics.

### PRESCRIPTIONS AND DISPENSING.

We were discussing the method of alternate additions of oil and of water in making emulsions, and instanced the officinal castor oil mixture, namely—

| | |
|---|---|
| ℞ Olei Ricini ... ... ... ... ... | ℥iij. |
| Mucilaginis Acaciæ ... ... ... | ℥jss. |
| Aquæ Aurantii Floris ... ... ... | ℥j. |
| Aquæ Cinnamomi ... ... ... ... | ℥ijss. |

as a case where this method was most useful. The castor oil should be added gradually to the mucilage in a mortar, with trituration, portions of the mixed waters being added alternately with the portions of oil; an excellent product results, far superior to the mixture that was formerly officinal, in which the emulsifying agent was alkaline, and the product consequently soapy.

Acacia gum is a very useful emulsifying agent for other substances besides fixed oils, and we shall consider certain of these before passing on to other emulsifiers. There is another of the officinal mixtures in which this gum is ordered, namely Mistura Amygdalæ Composita. The real substance to be emulsified here is the fixed oil contained in the almonds, and as the latter also contain some gum, an emulsion may be formed by simply beating them with water; but the quantity of gum is insufficient to retain the oil permanently emulsified, and further gum is therefore ordered. The mixture is mainly employed as a pleasant vehicle for insoluble drugs. If the almonds are well ground with the sugar and gum their whole substance becomes well subdivided, and as the oil is already in minute globules in the seed, the conditions are very favourable for preparing an emulsion, and an excellent result is obtained.

Liquid extract of male fern differs both in mode of preparation and in nature from other liquid extracts, which are for the most part like either very strong tinctures or solutions of solid extract in water or spirit. In the case of male fern, however, the liquid extract is made by percolation with ether, and on distilling off the solvent, an oily thick liquid remains. This is not miscible with water, and when prescribed as a draught or mixture it must be dispensed as an emulsion. Several different emulsifying agents may be used, gum acacia being one of them; a larger proportion is necessary than in the case of fixed oils.

| | |
|---|---|
| ℞ Extracti Filicis liquidii ... ... ... ... | ʒij. |
| Mucilaginis Acaciæ ... ... ... ... | ʒij. |
| Aquam Cinnamomi ... ... ... ad | ℥ij. |

The extract should be poured on to the mucilage in a mortar or measure, and the two mixed by stirring with the pestle or a glass rod; the cinnamon water is then added in small quantities at a time, each addition being mixed in by trituration. Another method is to mix the liquid extract of male fern with a drachm of powdered gum acacia in a mortar, and then to add 3 drachms of cinnamon water in one quantity, the whole being triturated till an emulsion is formed; the rest of the water may then be added. This liquid extract, however, may be made into a better emulsion with some of the other agents. A little practice with male fern extract will soon impress upon one the fact which should always be borne in mind in making any emulsion in a mortar, that what is required is *rapid light action of the pestle, and not pressure by any means.*

Copaiba, often incorrectly termed a balsam, is really an oleo-resin, and both the essential oil and the resin, of which it consists, require to be emulsified if the drug is to be administered in the form of a mixture. A satisfactory result can be obtained by gum acacia, although other agents are often ordered. In the following—

| | |
|---|---|
| ℞ Copaibæ ... ... ... ... ... ... | ℥ss. |
| Mucilaginis Acaciæ ... ... ... | ℥ss. |
| Spiritus Etheris Nitrosi ... ... ... | ʒij. |
| Aquam ... ... ... ... ad | ℥iv. |

first put the mucilage into the mortar, add a small quantity of the copaiba, and mix well; continue the addition of small quantities, adding also a little water from time to time, so as to keep the mixture of the right consistency for working; when all the copaiba has been added, and the emulsion is milky, transfer to the bottle and make up to 3½ ounces; dilute the spirit with an equal volume of water and add it last. Another useful prescription, often given to patients with dropsy on account of its diuretic properties, is the following:—

| | |
|---|---|
| ℞ Copaibæ Resinæ ... ... ... | gr. xv. |
| Alcohol (90 per cent.) ... ... ... | ♏xx. |
| Pulveris Tragacanthæ Compositi ... | gr. xv. |
| Syrupi Zingiberis ... ... ... | ʒj. |
| Aquam ... ... ... ... ad | ℥j. |

Warm the copaiba resin with the alcohol, triturating all the time, until a thick homogeneous liquid is obtained; rub the compound tragacanth powder into a thick cream with a little of the water in a mortar; pour the copaiba and alcohol into the latter in successive portions, stirring well; add the rest of the water, and put in the syrup of ginger last of all.

Cod-liver oil may be emulsified by means of a variety of agents, and, generally speaking, gum acacia is not so good as some others for the purpose; when it is desired, however, to give a quantity of some other medicaments simultaneously, it is better to trust to acacia. Suppose, for example, one wished to give

| | |
|---|---|
| ℞ Olei Morrhuæ ... ... ... ... | ℥ijss. |
| Syrupi Ferri Iodidi ... ... ... | ℥ij. |
| Aquam Chloroformi ... ... ad | ℥viij. |

it will be necessary to include 4 drachms of gum acacia for the emulsification of the oil. The gum should first be made into a mucilage by stirring with half an ounce of the chloroform water, a few minutes being allowed for solution to be complete. The oil should then be added in small quantities with constant trituration, a little chloroform water being added at intervals whenever the mixture is becoming too thick. The emulsion is made up to 6 fluid ounces, and the syrup of iodide of iron, diluted with the rest of the chloroform water, should be poured in last.

(*To be continued.*)

